# Out-of-Pocket Costs for Facility-Based Obstetrical Care in Rural Guatemala

**DOI:** 10.5334/aogh.3223

**Published:** 2021-08-02

**Authors:** Michel Juarez, Kirsten Austad, Peter Rohloff

**Affiliations:** 1 Center for Research in Indigenous Health, Wuqu’ Kawoq | Maya Health Alliance, 2a Avenida 3-48 Zona 3, Barrio Patacabaj, Tecpán, Chimaltenango, Guatemala; 2 Department of Family Medicine, Boston Medical Center, 1 Boston Medical Center Place Boston, MA, USA; 3 Division of Global Health Equity, Brigham and Women’s Hospital, 75 Francis Street, Boston, MA 02115, USA

## Abstract

**Background:**

Rural Indigenous Maya communities in Guatemala have some of the worst obstetrical health outcomes in Latin America, due to widespread discrimination in healthcare and an underfunded public sector. Multiple systems-level efforts to improve facility birth outcomes have been implemented, primarily focusing on early community-based detection of obstetrical complications and on reducing discrimination and improving the quality of facility-level care. However, another important feature of public facility-level care are the out-of-pocket payments that patients are often required to make for care.

**Objective:**

To estimate the burden of out-of-pocket costs for public obstetrical care in Indigenous Maya communities in Guatemala.

**Methods:**

We conducted a retrospective review of electronic medical record data on obstetrical referrals collected as part of an obstetrical care navigation intervention, which included documentation of out-of-pocket costs by care navigators accompanying patients within public facilities. We compared the median costs for both emergency and routine obstetrical facility care.

**Findings:**

Cost data on 709 obstetric referrals from 479 patients were analyzed (65% emergency and 35% routine referrals). The median OOP costs were Q100 (IQR 75–150) [$13 USD] and Q50 (IQR 16–120) [$6.50 USD] for emergency and routine referrals. Costs for transport were most common (95% and 55%, respectively). Costs for medication, supply, laboratory, and imaging costs occurred less frequently. Food and lodging costs were minimal.

**Conclusion:**

Out-of-pocket payments for theoretically free public care are a common and important barrier to care for this rural Guatemalan setting. These data add to the literature in Latin American on the barriers to obstetrical care faced by Indigenous and rural women.

## Introduction

Guatemala is the most populous country and largest economy in Central America and is classified by the World Bank as an upper-middle-income country [1,2]. However, Guatemala is also a country of extreme disparities between urban centers versus rural communities, which are largely Indigenous Maya. For example, 64% of the rural population versus 34% of the urban population lives below the national poverty line [3]. In addition to economic disparities, rural Indigenous Maya communities in Guatemala have some of the worst obstetrical and neonatal health outcomes in Latin America [4,5]. The national maternal mortality rate is 113 per 100,000 live births compared to 155 for the Indigenous population [5].

One factor contributing to these poor health outcomes is that many Indigenous women have a strong preference for home birth with the assistance of a traditional midwife, with only 50.3% giving birth in a healthcare facility compared to 82.1% of the non-Indigenous population [6]. Reasons for this include the central cultural and spiritual role of traditional midwives in Maya healthcare, but also language barriers for speakers of Mayan languages accessing biomedical healthcare facilities and widespread experiences of discrimination and disrespectful or abusive biomedical care [7,8]. To address these barriers and improve facility birth rates for high-risk pregnancies, several interventions have been investigated, include the use of maternity homes, mobile health technology, care navigation, and rights-based auditing and accountability initiatives [9,10,11,12,13].

An additional barrier in facility-level obstetrical care is the cost of care, which is frequently mentioned in qualitative studies with Indigenous women and their families [7,8,14,15]. Despite a universal mandate guaranteeing free public healthcare, in reality this is rarely the case. Guatemala’s public healthcare system is chronically underfunded and plagued by corruption. As a result, frontline facilities are often stocked-out of essential medications or lack basic equipment (e.g. ultrasound machines) and patients are required to pay out of pocket (OOP) for essential medications, supplies and tests at third-party facilities that proliferate in close proximity to public facilities [12,13,16,17,18]. Additional sources of expense to women seeking obstetrical care include transportation to facilities and food and lodging. Coupled with very limited availability of health insurance [16,17,18], OOP spending on healthcare is estimated to represent more than 50% of all national financing for “free” public sector healthcare [18].

Unfortunately, despite the central nature of cost in Indigenous patients’ experiences of healthcare, data on out-of-pocket spending for obstetrical care in rural Guatemala remain largely qualitative [7,8,14,15]. The unpredictable nature of obstetrical emergencies and low health literacy and numeracy of many rural Indigenous Maya women have made quantifying cost data challenging. An additional documentation challenge is that informal payments (cash exchange without receipts) by individual users to pharmacies are prevalent as a work-around to avoid payment of value-added taxes. Obtaining quantitative data on the cost of obstetrical care in Guatemala is important, as it has significant implications for health advocacy initiatives through specifying the magnitude of the problem and its financial implications for patients.

Studies on OOP spending are notably absent from Latin America, despite it being the most economically unequal region of the world [19]. However, studies from a range of public facilities in countries like Pakistan, India, Madagascar, Tanzania, Burkina Faso have documented significant OOP spending for obstetrical care in public facilities ostensibly providing free care, particularly related to spending on medications, laboratory and imaging studies, and consumable supplies either not provided, stocked-out, or illegally charged by facilities. Other documented costs include those related to transportation and informal payments to providers or facilities [20,21,22,23,24,25,26]. In many occasions these OOP expenditures rise to the level of catastrophic health expenditures [24,27,28].

Recently we implemented and evaluated a care navigation strategy to improve facility-level care for Indigenous women [11]. As part of this intervention, traditional midwives identified patients from rural villages needing facility-level care and referred these patients to a care navigator/advocate, who then accompanied the patient through all phases of their care. Care navigators were trained to carefully document multiple process indicators related to the referral process, including all OOP payments. This project therefore represents a unique opportunity to quantify OOP costs of obstetrical care in rural Guatemala, and we present these findings here.

## Methods

### Study Context

The study was a retrospective chart review of electronic data on obstetrical referrals collected as part of an obstetrical care navigation intervention, where primary clinical outcomes have already been reported [11]. Data were collected in collaboration with Maya Health Alliance, a primary care organization providing health services to rural communities in five Guatemalan provinces. The intervention occurred in a single health district (Tecpán, Guatemala) with 41 collaborating traditional midwives attending approximately 750 births/year. All facility referrals and care were provided by the Tecpán health district’s secondary hospital or the provincial tertiary hospital (province of Chimaltenango). The study was approved by the Institutional Review Boards at Brigham and Women’s Hospital and Maya Health Alliance. More than 95% of the population of Tecpán self-identifies as Indigenous Maya, and more than 65% live in poverty [29].

### Study design and key variables

Obstetrical care navigators accompanying patients during facility level care documented OOP costs in major subcategories, including medications and consumable supplies, laboratory and imaging studies, transportation, and food and lodging. All data were documented within Maya Health Alliance’s electronic health record (EHR, OpenMRS 1.9.9, *www.openmrs.org*) for each patient encounter. Patient-level data and data on facility level referrals were then extracted from the EHR using an SQL query procedure and tabulated.

We analyzed total cost and cost by major subcategories for each episode of facility-level care, including all such episodes for patients with multiple facility-level encounters. We separately analyzed costs for emergency and routine referral encounters. Emergency referrals were defined as urgent obstetrical indications requiring immediate facility-level care (e.g., hemorrhage, severe pre-eclampsia). Routine referrals were defined as encounters for non-urgent ambulatory matters where care in a facility rather than community setting was indicated (e.g., gestational diabetes). For medication/supply and laboratory/imaging OOP costs, we reviewed itemized receipts (e.g., giving the names of individual tests or medications) collected by care navigators, where these were available, although many vendors did not provide itemized receipts. When itemized receipts were not available, analysis of cost was limited to categorical costs documents in the electronic forms by navigators.

### Statistical analyses

We performed all analyses using Stata version 14 (College Station, Texas). We compared the distribution of cost data for emergency and routine referrals, as well as for major cost subcategories using the Wilcoxon rank-sum test. Violin plot to visualize the density distribution of costs were generated using R version 3.4.1 (Vienna, Austria).

## Results

**Figure 1:**
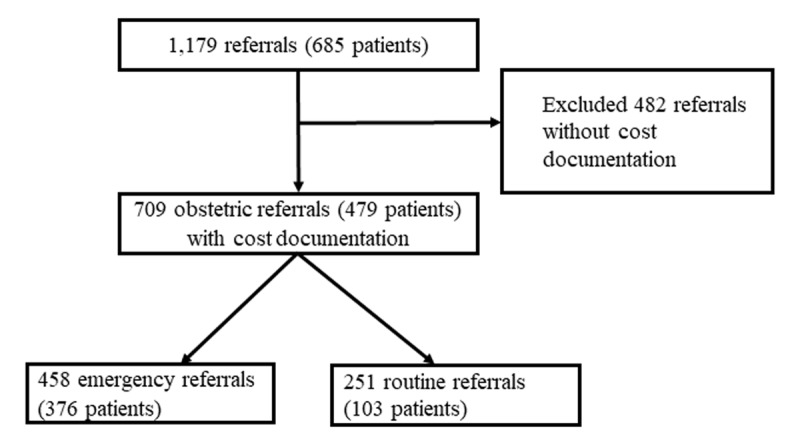
Schematic of chart review process and data included in analysis.

We reviewed all available obstetrical records in the EHR from March 1, 2017 (the beginning of the care navigation intervention) to May 31, 2019. We identified 1179 referrals from 685 unique patients generated by traditional midwives to facility-based care during this time frame. Of these 482 lacked documentation of cost data. Therefore, cost data from 709 referrals (479 patients) were included in this analysis (458 emergency referrals, 251 routine referrals) (***Figure 1***).

### Demographic and clinical characteristics for patients receiving facility-based care

***Table 1*** summarize salient characteristics of women included in the analysis. The median age was 27 years, and 43% were primiparous. Among those with a prior birth, median parity was two and 37% had received some facility-based care in their last pregnancy. In terms of outcomes for the index pregnancies include in this analysis, 36% gave birth in a healthcare facility with the remaining giving birth at home. Among those with a facility birth, more than one-third were by caesarean section. There were 11 stillbirths (1.6%), 20 neonatal deaths (2.9%), and no maternal deaths in the analysis period.

**Table 1:** Characteristics of Patients Receiving Facility Level Care.


CHARACTERISTIC^1^	VALUE

Age, years: median [IQR] n = 675	27 [23–33]

Parity, births: median [IQR]^3^ n = 475	2 [1–4]

Nulliparous, %	39

Facility-level care in last pregnancy, %, n = 252^2^	61

Facility delivery, %, n = 631	74

Caesarean delivery, %	38

Stillbirth, %, n = 682	1.6

Neonatal death, %, n = 682	2.9

Maternal death, %, n = 682	0


^1^ Missing data for individual items indicated by giving denominator for each item as indicated. ^2^ Excludes nulliparous women.

### Out-of-pocket costs associated with receiving facility level care

A total of 709 referrals had documented costs and were included in the cost analysis (65% emergency and 35% routine referrals). The median OOP costs were Q100 (IQR 75–50) [$13 USD] and Q50 (IQR 16–120) [$6.50 USD] for emergency and routine referrals respectively (***Table 2***, p < 0.001 for difference in cost between category). Transportation expenditures were the most frequently documented cost category (95% and 55% of emergency and routine referrals, respectively), and transportation costs for emergency referrals were significantly more expensive (p < 0.001). Medication and supplies costs were incurred in 15% of all referrals. Laboratory and imaging costs were incurred in 7% of emergency and 27% of routine referral, and food and lodging costs were incurred in 2% of emergency and 29% of routine referrals. For these three costs categories, median costs were similar for emergency and routine referrals (***Table 2***). Finally, 63 patients (13%) required more than one obstetrical referral and incurred a median OOP cost over the entire pregnancy for care of Q300 [IQR 190–484].

**Table 2:** Out of pocket costs by expense category and referral type.


COST CATEGORY^1^	EMERGENCY REFERRALS	ROUTINE REFERRALS	P VALUE^2^

Total cost, median [IQR], n with documented costs	100 [75–150], n = 458	50 [16–120], n = 251	<0.001

Medications and Supplies, median [IQR] (% with costs)	63 [30–100] (15%)	51 [20–95] (15%)	0.57

Laboratory and Imaging, median [IQR] (% with costs)	120 [85–179] (7%)	120 [86–120] (27%)	0.43

Transportation, median [IQR] (% with costs)	100 [60–150] (95%)	28 [15–60] (55%)	<0.001

Food and Lodging, median [IQR] (% with costs)	25 [18–37.5] (2%)	15 [10–30] (29%)	0.17


^1^ Cost data given in Guatemalan quetzales (1 quetzal = 0.13 US dollar). For each category, costs are given for all referrals with documented OOP spending. ^2^ Wilcoxon rank-sum test.

**Figure 2:**
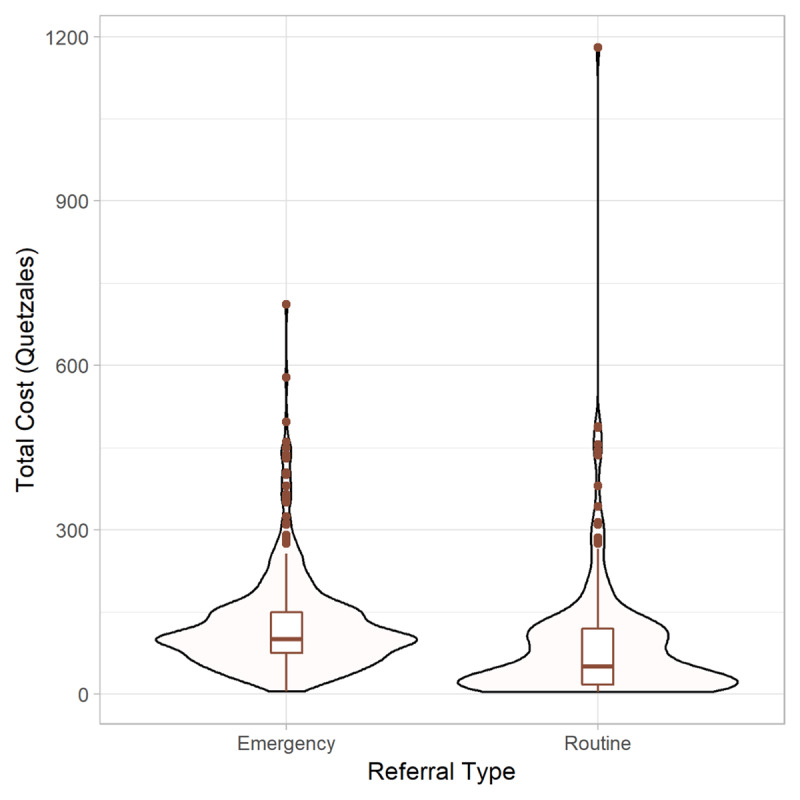
Violin plots of the distribution of out-of-pocket costs in quetzales for both emergency and routine facility referrals. 1 quetzal = 0.13 US dollar.

The distribution of OOP costs was visualized using violin plots (***Figure 2***). A bi-modal distribution was observed in routine referrals in comparison with emergency referrals, with many more low-cost expenditures observed. However, at the same time, the highest expenditures (outliers) were reported among routine referrals.

**Figure 3:**
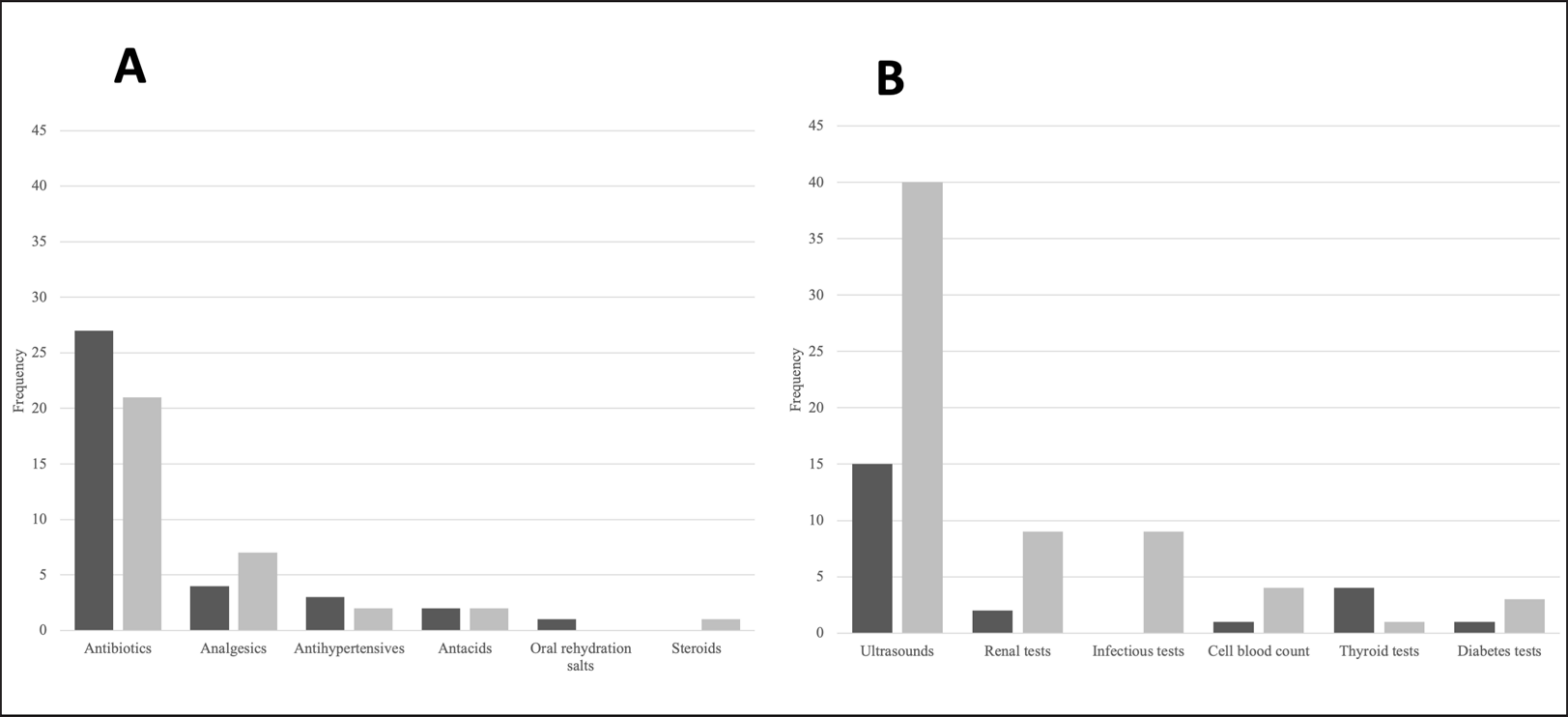
Out-of-pocket payments for major categories of medications/supplies **(A)** and imaging/laboratory studies **(B)**. Raw frequencies of out-of-pocket payments for each category are given separately for emergency referrals (dark bars) and routine referrals (light bars).

We explored the subcategories of medication/supply and laboratory/imaging costs by categorizing the most five most frequently occurring items that individual patients purchased OOP when detailed itemized purchase receipts were available (***Figure 3***). Itemized receipts were available for 114 observations (45 medication expenditures and 69 laboratory expenditures). For both emergency and routine referrals, the most commonly purchased OOP medications and supplies were antibiotics and analgesics (***Figure 3A***). There were 69 total instances of emergency OOP costs (15% of all emergency referrals) and 38 total instances of routine OOP costs in this category (15% of all routine referrals).

For the laboratory/imaging categories, the most common OOP costs for emergency referrals were obstetrical ultrasound examinations and laboratory assays for renal and thyroid function. On the other hand, for routine referrals, the most common costs were for obstetrical ultrasound examinations, renal function tests, and prenatal infectious serological workups (***Figure 3B***). There were 30 total instances of emergency OOP costs (6.6% of all emergency referrals) and 67 total instances of routine OOP costs in this category (27% of all routine referrals).

### Sensitivity analysis

A significant proportion of referrals (41%, 482/1179, ***Figure 1***) did not have any cost data documented by care navigators during their facility encounters. We explored for systematic differences between individuals with and without reported cost analysis (Supplementary Table 1). Individuals with and without reported costs data did not differ on basic clinical characteristics. Facility birth rates were higher in those with cost data (p = 0.01). Neonatal mortality was higher in those with cost data, although this was not statistically significant (p = 0.05).

## Discussion

We examined the OOP costs for emergency and routine obstetrical care in public health facilities in rural Guatemala. Given that rural—and especially Indigenous Maya—communities in Guatemala have some of the worst obstetrical outcomes in Latin America, significant attention has been paid to factors contributing to these poor outcomes, including cultural and language barriers, low-quality health services, and disrespectful or discriminatory practices. Anecdotally, the cost of care due to illegal user fees or supply stock-outs in public facilities is also a major concern but has been difficult to quantify. We took advantage of detailed cost data collected for obstetrical facility-level referrals as part of an obstetrical care navigation intervention to quantify these costs [11]. These data contribute to ongoing discussions within Guatemala on right to health, corruption, and healthcare financing, where OOP spending now represents more than 50% of national healthcare spending [18]. They also provide a Latin American perspective on OOP costs for public obstetrical services, which has been better documented in Africa and Asia [20,21,22,23,24,25,26].

This analysis should also be framed against the significant burden of poverty in rural Guatemalan communities. In Tecpán, Guatemala where this study took place, more than 65% of the population lives in poverty [29]. Most are subsistence farmers, with limited and sporadic cash flow. In prior work, we have shown that average expenditures are around $5 USD per capita per week, which are well below the expenditure required to ensure a nutritionally complete diet [30]. Furthermore, for those who earn cash wages, more than 70% are employed in the informal economy and earn considerably less than the minimum wage of $11.50 USD per day [31]. These figures highlight the difficulties much of the population faces meeting basic subsistence needs and vulnerability to other unanticipated spending, such as the OOP spending for theoretically free public healthcare that we document here [31,32].

We analyzed cost data from more than 700 instances of facility-level obstetrical care, including both emergency and routine care. Of these the median OOP costs were roughly $13 USD and $6.50 USD for emergency and routine referrals respectively (***Table 2***). Transportation expenditures were the most frequently documented cost category, occurring in 95% and 55% of emergency and routine referrals, respectively. Medication/supplies and laboratory/imaging costs were also frequent. Taken in context with the income and household spending parameters discussed above, it is apparent how OOP costs are a significant deterrent to seeking facility-based obstetrical care for rural Guatemalans. For example, OOP costs for a single obstetrical emergency represents more than one week of food-related spending for two adults or more than one day’s wages at minimum wage. These costs represent direct OOP spending on medical care or seeking care (food, lodging), and do not take into account other unmeasured accrued costs such as childcare and lost wages.

Most notable is the prevalence of OOP spending on transportation. In recent years, as part of national policy efforts to improve access to emergency transportation, the Ministry of Health, the Executive Branch, and local municipal governments have all invested in installing and staffing local ambulance services [33]. As evidence of the efforts, virtually all referrals included in our analysis here were transported to facilities by public ambulance services. At the same time, however, ongoing corruption and budgetary misappropriations prevail, and so salary payments to ambulance staff are irregular and ambulance teams frequently do not have funds to purchase fuel or perform vehicle maintenance. As a result, informal and illegal “co-pays” from all patients are demanded to cover these costs [34]. As we document here, these ambulance co-pays represent the bulk of OOP costs for obstetrical care.

In addition to providing an estimate of overall and category-specific OOP costs for obstetrical care, itemized receipts were available for a proportion of referrals. Analysis of these receipts allowed us to examine the prevalence and distribution of specific OOP costs for medical care. Collection of these receipts by the program care navigators is a unique opportunity, since although patients routinely are required to purchase many essential medications and supplies in theory supplied by public hospitals, there is limited published data available on where these specific costs fall [35]. Our data demonstrate (***Figure 2***) that antibiotics were the most significant medication/supply-related OOP cost for both emergency and routine obstetrical care. In addition, the lack of access to obstetrical ultrasound within an obstetrical facility is astonishing, as is the frequency of charges for common prenatal laboratory testing (infectious disease serologies, kidney function), both of which were often paid OOP by patients to third-party vendors located outside hospital facilities.

This brief report has multiple limitations. First, as we have previously reported, the care navigation intervention provided accompaniment services to around 40% of all pregnancies within the catchment area [11], primarily those with more severe pregnancy-related complications or complex needs. As such, our data here does not capture OOP costs related to unaccompanied self-referral to care and, therefore, likely represents an upper limit on the OOP costs incurred. Given the structure of the data collection procedures, only direct OOP costs were captured and estimates of other incurred costs, such as lost wages, were not assessed. In addition, cost data was not collected by care navigators for all referrals, although our sensitivity analysis did not detect marked demographic or clinical differences between those patients with or without documented costs (Supplementary Table 1). Given the prevalence of informal payments, itemized receipts were available only for a limited number of referrals and, therefore, the analysis of common stocked-out items may not be representative (***Figure 3***). Finally, additional qualitative work is needed to understand individuals’ perceptions of these costs and ability to pay, work which is ongoing now at our institution.

## Conclusions

We used a unique data set on public sector obstetrical care to estimate the OOP costs for theoretically free public care in a rural Guatemalan setting. We found that OOP costs for ambulance transport were highly prevalent and that OOP costs for medications/supplies and laboratory/imaging studies were also common, especially for antibiotics, obstetrical ultrasound, and routine prenatal laboratory testing. This data add to the literature in Latin American on the barriers to obstetrical care faced by Indigenous and rural women.

## Additional File

The additional file for this article can be found as follows:

10.5334/aogh.3223.s1 Supplementary Table 1. Characteristics of Patients Receiving Referrals By Absence of Documented Cost Data.
