# Supplementary material for: Out-of-Pocket Costs for Facility-Based Obstetrical Care in Rural Guatemala
Source: Ann Glob Health. 2021 Aug 2;87(1):75. doi: 10.5334/aogh.3223 (PMC8344954; doi:10.5334/aogh.3223)
Supplement: Supplementary Table 1. — Characteristics of Patients Receiving Referrals By Absence of Documented Cost Data. [file agh-87-1-3223-s1.pdf]

Supplementary Table 1. Characteristics of Patients Receiving Referrals By Absence of Documented Cost Data

Characteristic[1]	With Cost Data	Without Cost Data	P Value[2]
Age, years: median [IQR] (n)	27 [23-33] (474)	26 [22-33] (201)	0.19
Parity, births: median [IQR] (n)[3]	2 [1-4] (205)	2 [1-4] (87)	0.77
Nulliparous, % (n)	40 (340)	35.56 (135)	0.15
Facility-level care in last pregnancy, % (n)[3]	38 (316)	33 (103)	0.36
Facility delivery, % (n)	78 (445)	63 (186)	0.01
Caesarean delivery, % (n)	40 (445)	34 (186)	0.08
Stillbirth, % (n)	1.5 (477)	2.0 (205)	0.65
Neonatal death, % (n)	3.8 (477)	1.0 (205)	0.05
Maternal death, % (n)	0	0	n/a

[1]Missing data for individual items indicated by giving denominator for each item as indicated. [2]Rank-sum test for continuous variables, chi-square test for categorical variables. [3]Excludes nulliparous women
